# Male Hysteria: Main Psychopathological Theories and Clinical Presentations (Narrative Literature Review)

**DOI:** 10.1192/j.eurpsy.2025.1917

**Published:** 2025-08-26

**Authors:** S. Elloumi, S. Kolsi, A. Bouaziz, W. Abbes

**Affiliations:** 1 Psychiatry, University hospital, Gabes, Tunisia

## Abstract

**Introduction:**

Hysteria has been historically linked primarily to women; however, its importance in men has recently gained attention. This is why we found it interesting to study male hysteria. Therefore, understanding psychopathological theories offers a valuable framework for highlighting its clinical characteristics.

**Objectives:**

Describe the main psychopathological theories of male hysteria and its key clinical specifications referring to the theories.

**Methods:**

We conducted a narrative literature review of articles and thesis published between 2009 and 2022, using databases such as PubMed, Google Scholar, and HAS (Haute Autorité de Santé) with predefined keywords such as “men”, “hysteria”, “masculine” and “conversion”. This review culminated in a narrative synthesis that aims a cohesive discussion integrating key findings from the literature.

**Results:**

The bibliography first distinguished between two pairs in order to define masculine hysteria: Anatomical (male/female) and psychological (feminine/masculine), highlighting that masculine hysteria can occur in both men and women. Therefore, a male may identify with either masculine hysteria or feminine hysteria, the latter of which can be related to male homosexuality.

Research indicates that masculine hysteria is associated with theories of sexual identity conflict, which involve the repression of feminine traits and fears of castration. Additionally, cultural norms often restrict men’s emotional expression, favoring strength over vulnerability, along with personal, relational, and environmental influences.

Clinical observations suggest that masculine hysteria typically presents with physical symptoms and dramatic expressions. The type and intensity of these symptoms may vary based on different early life experiences.

**Conclusions:**

Referring to literature, the specific clinical signs leading to a diagnosis of men hysteria seem to be difficult to identify and not well developed. We also concluded that hysteria is too much related to lived gender rather than anatomical gender.

**Disclosure of Interest:**

None Declared

